# Influence of Rolling Direction on Barkhausen Noise in Low-Alloyed Steel MC500

**DOI:** 10.3390/ma19030576

**Published:** 2026-02-02

**Authors:** Radoslav Koňár, Branislav Vavák, Mária Čilliková, Katarína Zgútová, Miroslav Neslušan, Jaroslav Odrobiňák

**Affiliations:** 1 Faculty of Mechanical Engineering, University of Žilina, Univerzitná 1, 010 26 Žilina, Slovakia; radoslav.konar@fstroj.uniza.sk (R.K.); maria.cillikova@fstroj.uniza.sk (M.Č.); 2 Slovak Railways, R&D Institute of Railways, 010 01 Žilina, Slovakia; vavak.branislav@zsr.sk; 3 Faculty of Civil Engineering, University of Žilina, Univerzitná 1, 010 26 Žilina, Slovakia; katarina.zgutova@uniza.sk (K.Z.); jaroslav.odrobinak@uniza.sk (J.O.)

**Keywords:** Barkhausen noise, steel MC 500, tensile test, rolling direction, plastic straining

## Abstract

This study examines the impact of rolling direction on Barkhausen noise emission from the low-alloyed steel MC 500 during a uniaxial tensile test. The samples of gauged shape were cut along both the rolling and transverse directions to investigate the process of magnetic anisotropy alterations, as expressed in terms of Barkhausen noise and the extracted features. Barkhausen noise was studied as a function of both elastic and plastic straining (up to plastic strain 21.5%), and the role of domain wall realignment with respect to the rolling direction, as well as the direction of the tensile load, was analysed. Barkhausen noise emission is linked to both the stress state and the microstructure, and the role of external stressing is contrasted with the residual stress state. Barkhausen noise is measured directly during a tensile test (in situ) as well as after unloading (ex situ). It was found that Barkhausen noise is significantly affected by stress directly during the tensile test (in situ), whereas the contribution of residual stresses is less pronounced. Barkhausen noise measured in situ during the tensile test in the direction of the tensile load is higher (about 1100 mV) compared to the transverse direction (about 500 mV). However, this relationship is reversed for the ex situ measurements, especially for the more developed plastic strains above 15%. The influence of rolling direction on Barkhausen noise is relatively minor, and Barkhausen noise after matrix yielding is primarily affected by increasing dislocation density growing from 3 × 10^15^ up to 5 × 10^15^ m^−2^.

## 1. Introduction

Barkhausen noise is a well-known physical phenomenon originating from the motion of irreversible and discontinuous domain walls (DWs) in ferromagnetic bodies [1,2,3]. DWs are kept in their position under an altering magnetic field due to the presence of pinning sites, and their motion is suddenly initiated as soon as the magnetic field attains the pinning strength of the pinning sites. During their motion, DWs emit both acoustic and electromagnetic pulses. The latter is usually abbreviated as MBN (magnetic Barkhausen noise). DWs, during their motion, encounter all lattice imperfections (dislocation tangles, precipitates, non-ferromagnetic phases, grain boundaries, etc.) and therefore contain information about the microstructure [4,5,6,7,8]. Moreover, MBN is often explained in terms of stress state with respect to magneto-elastic coupling [9,10]. MBN is frequently used for monitoring components in real-world industries, especially due to their altered matrix [11,12], or after surface hardening [13,14]. Furthermore, this technique can be employed for characterising ferromagnetic bodies after their hot or cold forming, heat treatment, and chemical treatment, among other applications [15,16,17].

The MBN technique was also employed for monitoring components exposed to uniaxial tension, and MBN was investigated in both the elastic regime and beyond the yield point [18,19,20]. Apart from other aspects, these studies also reported alterations in the easy and hard axes of magnetisation. It was reported in our previous studies [18,19] that DWs after yielding tend to realign in the direction perpendicular to the tensile stress (S235 and Trip steels). A similar behaviour was reported by Lindgren and Lepistö [21], as well as by Roskosz et al. [22]. However, Schmidova et al. [23] found reversed evolution for the interstitial free steel loaded in a direction transverse to the rolling direction. Moreover, some studies have reported that MBN can grow after cold forming, despite an increase in dislocation density [15,23,24], in contrast to other studies [18,19].

Magnetic anisotropy expressed in terms of MBN can often be detected, and the MBN technique is a promising tool for monitoring crystallographic texture in a fast and reliable manner [25]. Wang et al. [26] noted that cold rolling developed Goss or an α-fibre texture, resulting in strong angular MBN dependence. The preferential crystallographic orientation in oriented electrical steels, and the corresponding texture, can also be detected by macroscopic magnetic properties and the extracted MBN features [27]. Also, He et al. [28] noted that DW unpinning is valuably altered when remarkable crystallographic orientation is developed. Rolling direction takes a strong role in the phase transitions of austenite into martensite after shot peening, when stronger MBN emission can be measured along the sheet rolling direction at the expense of a less affected transversal one [29]. Krause et al. [30] proposed a model for the extraction of isotropic as well as anisotropic components of MBN as the feature for analysis of MBN angular dependence. Short-time Fourier transform (STFT) decomposes MBN into time-localised frequency components, and this post-processing also enables localization of specific Barkhausen events [31] when magnetic anisotropy is investigated. And finally, it was demonstrated that the degree of magnetic anisotropy is valuably affected by microstructure heterogeneity due to the altered conditions of hot rolling [32].

The history of sheets with respect to their rolling direction seems to have an influence on DW realignment (especially in the case of ex situ regime measurements), and this aspect has not been investigated yet. For this reason, this study investigates the influence of rolling direction on the MBN evolution in both in situ and ex situ regimes. The samples were exposed to uniaxial tension along the direction of previous rolling, as well as along the transverse direction. MBN was explained in terms of stress state (external or residual) as well as in terms of microstructure (especially dislocation density).

## 2. Materials and Methods

**Figure 1:**
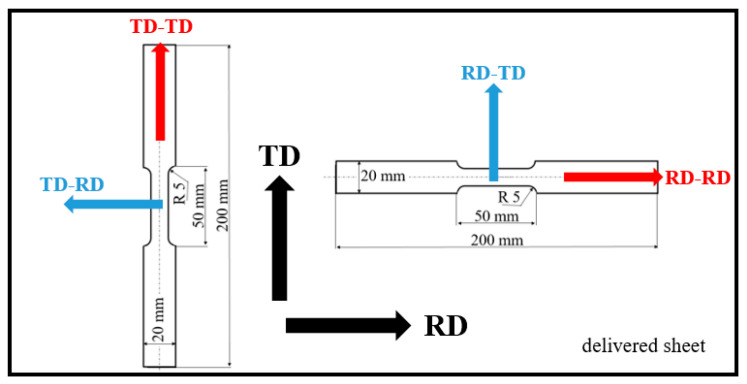
Brief illustration of the samples cut from the sheet with indicated directions.

**Figure 2:**
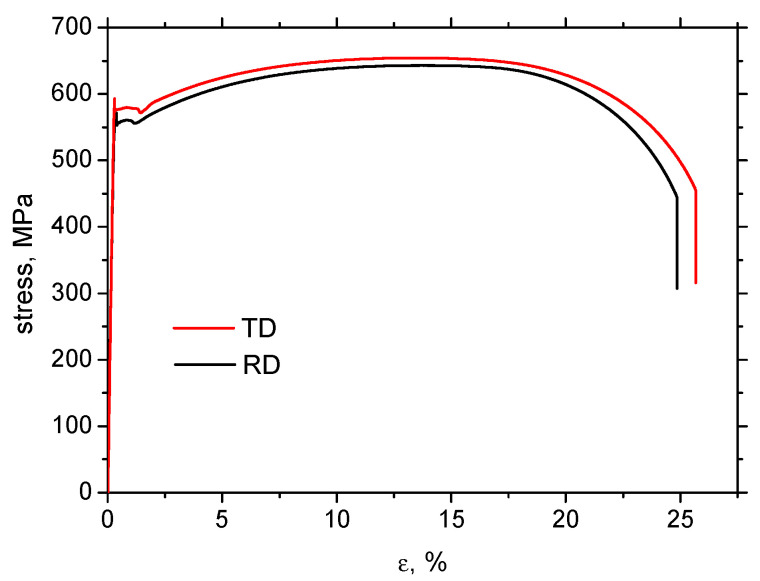
Stress–strain curves along RD and TD.

**Figure 3:**
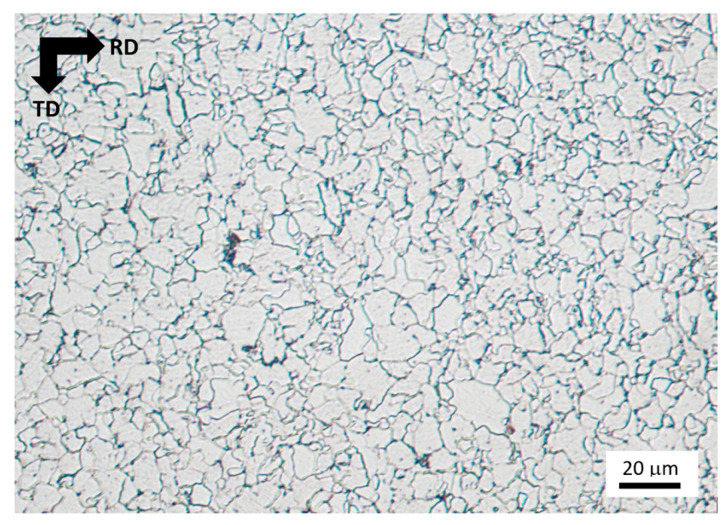
Metallographic image of MC500 microstructure in the as-received state, Nital 3%.

Samples for the tensile test were cut from a sheet with dimensions of 2000 mm in length, 1000 mm in width, and 5 mm in thickness. The sheet made of steel MC500 was hot-rolled above the austenitic temperature, followed by cooling using a medium cooling rate in order to attain the required mechanical properties and the corresponding fine-grained microstructure. The exact conditions of the hot rolling as well as the cooling are not known. Due to certain microstructure heterogeneity of the as-received sheet (especially near the sheet edges–based on the previous experience and applying the MBN technique [32]), the samples for the experiments were cut from the central region, and the regions near the edges with a size of about 100 mm were excluded from further processing. Furthermore, all samples before the uniaxial test were measured using MBN, and only those emitting similar values (within ± 5% of the average value) were employed for further experiments. The samples of the gauged shape were cut along the rolling direction (RD) as well as the transversal direction (TD), see Figure 1. Mechanical properties of the steel MC500 were obtained from the stress–strain curves illustrated in Figure 2, also see Table 1 (three repetitive measurements). Chemical composition of the investigated steel is indicated in Table 2. Metallographic observations were carried out on the specimens with a length of 16 and a width of 12 mm (routinely prepared by hot moulding, grinding, polishing, and 3% Nital etching for 5 s). The microstructure of MC500 is composed of fully ferritic grains with an average grain size of 6 μm (grain size obtained from the EBSD observation as reported earlier [33]) and a small fraction of pearlite islands, as shown in Figure 3.

Nomenclature with respect to abbreviated directions is listed in Table 3. Samples were cut and loaded along the RD and TD (see Table 3 and Figure 1) using uniaxial tension with an Instron 5985. True elastic strains were measured using the extensometer 2620-602 (Instron, Norwood, MA, USA) on a 25 mm length.

To obtain a true interpretation of MBN signals, conventional measurements of residual stresses and hardness (related to dislocation density) were also performed, along with observations of microstructural preferential orientation via light microscopy. Residual stresses and hardness measurements, as well as metallographic observations, were carried out ex situ on samples strained to predefined homogeneous plastic strains of 0.5%, 4.5%, 8.5%, and 12.5%, as well as in the region of plastic instability (necking) at 15.5%, 18.5%, and 21.5%. Three repetitive measurements were carried out for each *ε*.

Residual stresses were measured in all the aforementioned directions using the X-ray diffraction (XRD) technique (Proto iXRD Combo diffractometer (Proto Manufacturing Ltd., LaSalle, Ontario, Canada), *Kα*_1_ and *Kα*_2_ of {211} planes, Cr*Kα*, and the Winholtz and Cohen method, ½*s*_2_ = 5.75 TPa^−1^, s_1_ = −1.25 TPa^−1^). Evolution of *FWHM* of XRD patterns in this particular case is usually linked with dislocation density and the corresponding hardness of a body [34]. The dislocation density *δ* can be extracted from the XRD profiles when the full width at half maximum of XRD patterns is known:

(1) $$\delta =\;\frac{1}{D^{2}}\;$$

where *D* is the crystallite size linked with Equation (2):

(2) $$D=\;\frac{K.\lambda }{\beta .\cos\theta }$$

where K is the constant (0.89), *λ* is the X-ray wavelength, *β* is the FWHM of the XRD peak, and *θ* is the Bragg angle. Microhardness *HV1* (Vickers hardness applying load of 1 kg for 10 s, with 3 repetitive indents) was measured using the Innova Test 400TM device (INNOVATEST Europe BV, Maastricht, The Netherlands). 

MBN was measured in two different regimes. The in situ MBN measurements were carried out directly during the tensile test using a RollScan 350-magnetising voltage ± 5 V, magnetising frequency 125 Hz, sine profile, and sensor S1-18-12-01 (Stresstech Oy, Jyväskylä, Finland). Continuous MBN measurements were performed along the entire stress–strain curve using ViewScan software v4.0.0 CZ in the frequency range of MBN pulses, from 70 to 200 kHz. Only the effective value (rms) of the MBN signal was acquired and sampled at a frequency of 25 Hz. Further in situ measurements were carried out at predefined plastic strains using MicroScan software v5.4.1 (frequency range from acquired MBN pulses: 10 to 1000 kHz, with a sampling frequency of 6.4 MHz). Ex situ measurements were also carried out using MicroScan software on the samples strained on the predefined strain, the same as those measured during the in situ regime. Apart from the effective value of MBN emission (referred to as MBN), the Peak Position (*PP*) of MBN envelopes was analysed as well. *PP* refers to the position in the magnetic field in which the MBN envelopes reach the maximum. All MBN features presented in the paper represent the average value from 10 consecutive bursts (5 hysteresis cycles).

## 3. Results of Experiments and Their Discussion

### 3.1. Metallographic Observations and Microhardness Measurements

The history of thermomechanical treatment of the delivered sheet is unknown, but it can be assumed that the sheet was produced during hot rolling, followed by a cooling rate slow enough to avoid bainite transformation. Fine-grained microstructure and the corresponding mechanical properties are driven by the thermomechanical treatment as well as the contribution of a small volume of Ti and Nb. These alloying elements increase the density of nucleation sites during cooling, which, in turn, reduces the grain size. Moreover, Ti and Nb form small precipitates, strengthening the matrix as well [35,36].

**Figure 4:**
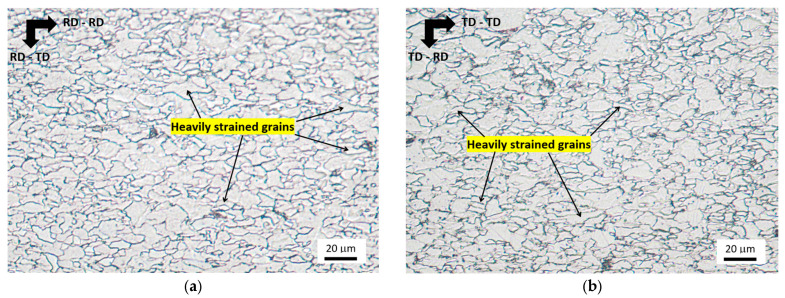
Metallographic images for *ε* = 21.5%: (**a**) cut along RD, and (**b**) cut along TD.

Due to uniaxial straining, the grains tend to become elongated along the direction of the tensile stress, as shown in Figure 4. Due to the small grain size, the degree of grain elongation is quite low, as contrasted with the previously reported values for the low-alloyed steel S235 [18]. Visible grain elongation for *ε* < 18.5% cannot be observed by visual observation, and no preferential elongation can be observed. The main reason can be attributed to the reduced distance for dislocation slip, which is linked to the low grain size and the corresponding formability of MC500. Visual observation of Figure 4 also indicates no valuable difference between the loading in uniaxial loading exerted along RD and TD. Figure 4 also demonstrates that the heavily strained grains along the direction of tension are neighbouring those being less affected. It is considered that the heavily strained grains are those that have a suitable crystallographic orientation against the direction of tensile stress. These grains are strained early beyond the yield strength, and their shape is altered within a quite wide range of plastic strains, as contrasted against the less elongated grains of the less suitable crystallographic orientation.

**Figure 5:**
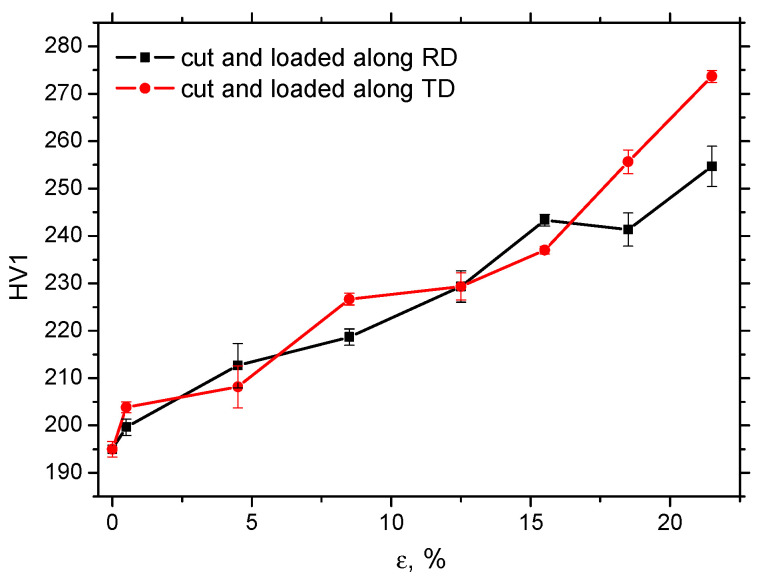
Evolution of *HV1* along *ε*.

The mechanism of strain hardening (see Figure 2) in this low-alloyed steel is primarily based on the increasing density of dislocations and their mutual interaction. The microhardness of MC500 is, therefore, mainly a function of dislocation density, which gradually and continuously increases, as shown in Figure 5. This Figure also demonstrates that the evolution of *HV1* along *ε* in RD and TD is nearly the same, especially in the region of homogeneous strains. However, a slightly higher *HV1* can be obtained beyond the necking in the case of samples cut and loaded in TD.

### 3.2. XRD Measurements

**Figure 6:**
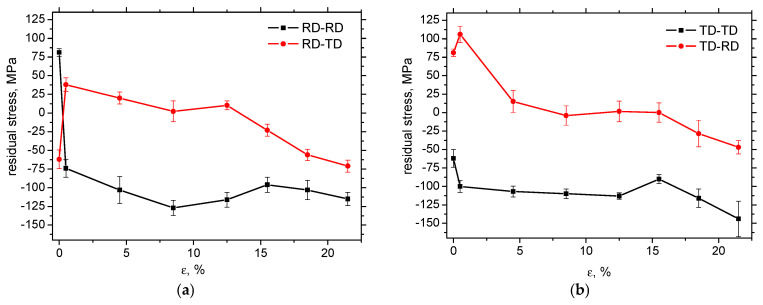
Evolution of residual stresses along *ε*: (**a**) cut along RD, and (**b**) cut along TD.

Surface tensile residual stresses of a magnitude of 80 MPa can be obtained in the RD on the samples before the tensile test, whereas the compressive ones of a magnitude of −62 MPa are obtained in the TD. Figure 6a illustrates that this stress anisotropy is reversed early after matrix yielding when the small residual tensile stresses measured perpendicularly to the direction of uniaxial tension are compensated by the compressive stresses in RD-RD. The tensile residual stresses tend to be shifted towards the compressive ones for the higher *ε*. Magnitude of the compressive stresses in RD-TD initially grows, but saturates early with respect to *ε*. The evolution of residual stresses in samples cut and loaded in TD is quite similar, apart from the initial reversal process for the lowest plastic strains, as shown in Figure 6b. Comparing Figure 6a,b, it can be reported that the evolutions are pretty similar.

**Figure 7:**
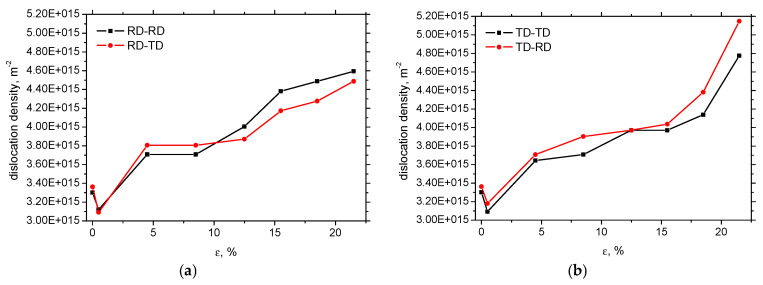
Evolution of dislocation density along *ε*: (**a**) cut along RD, and (**b**) cut along TD.

Figure 7 exhibits a similar evolution of the dislocation density of XRD as that illustrated in Figure 5. Apart from the gentle drop of the dislocation density early beyond the yielding (plastic deformation within the Lüders region [37]), the dislocation density gradually grows in all measured directions. The dislocation density in the region of homogeneous plastic strains is similar for the samples cut along RD and TD. Beyond the sample necking, an accelerated increase in the dislocation density is observed in the case of samples cut and loaded along the TD, as compared to samples cut and loaded along the RD. The evolution of the dislocation density in Figure 7 coincides with that depicted in Figure 5. It seems that the microstructural anisotropy developed during hot rolling has an influence on strain hardening beyond necking. However, a more profound insight into this aspect lies beyond the scope of this study.

### 3.3. Barkhausen Noise Measurements

**Figure 8:**
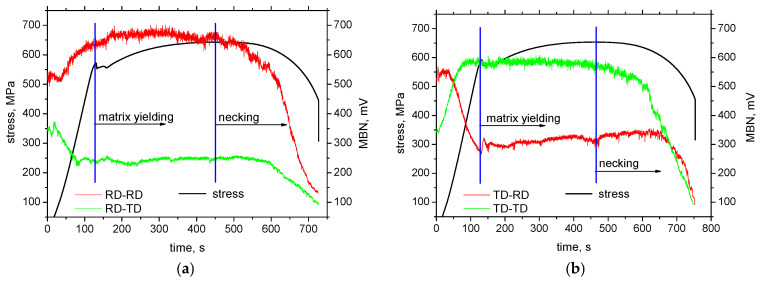
In situ MBN measurements using ViewScan (Stresstech Oy, Jyväskylä, Finland)—MBN in the frequency range from 70 to 200 kHz: (**a**) cut along RD, and (**b**) cut along TD.

The delivered sheet of MC500 exhibits certain magnetic anisotropy expressed in terms of MBN, when MBN in RD is more (about 525 mV) as compared with TD (about 350 mV), measured in the frequency range of MBN pulses from 70 to 200 kHz. Dynamic records of MBN emission in in situ tensile tests (see Figure 8) demonstrate the valuable influence of tensile stress. This Figure clearly indicates that MBN in the direction of tensile stress increases in the region of elastic stresses, compensated by a decrease in MBN in the perpendicular direction. For this reason, the magnetic anisotropy in the case of samples cut along TD is reversed early in the region of elastic strains, as shown in Figure 8b. The change in MBN in RD-RD and TD-RD is continuous within the elastic strains. However, the gradual drop of MBN in RD-TD, as well as the gradual growth in TD-TD, saturates before the matrix yields. Evolution of MBN versus stress state in the elastic region is driven by the competition between the energy of magnetocrystalline anisotropy *E_a_* and the magnetoelastic energy *E_σ_* [1,2,3]:

*E_a_* = *K*_1_(*α*_1_^2^*α*_2_^2^ + *α*_2_^2^*α*_3_^2^ + *α*_1_^2^*α_3_*^2^) *+ K*_2_(*α*_1_^2^*α*_2_^2^*α*_3_^2^) (3)

where *α*_1_, *α*_2_, and *α*_3_ are the direction cosines of the magnetisation vector and *K*_1_ and *K*_2_ are magnetocrystalline anisotropy constants.

*E_σ_* is valuably affected by magnetostriction [1,2,3]:

*E_σ_* = (−3*λ_s_cos*^2^*φ*)/2 (4)

where *λ_s_* is the magnetostriction of the matrix and *φ* is the angle between the direction of the magnetic field and the direction of loading stress *σ*.

Furthermore, the preferential alignment of grains (as illustrated in Figure 4) drops down the direction cosines at the higher strains (indicated in Equation (3)), which, in turn, eliminates the role of *E_a_*. On the other hand, *E_σ_* is not a function of the crystallographic orientation of the heavily elongated grains (see Equation (4)) and is more or less constant when the direction of the magnetising field is fixed. This effect contributes to the dropping MBN, especially at the higher strains. It can also be reported that *E_a_* is consumed earlier (*E_σ_* prevails over *E_a_* before the yielding) in TD due to the crystallographic anisotropy developed during hot rolling. This physical behaviour explains the earlier saturation of MBN measured in RD-TD and TD-TD.

The low degree of MBN alterations can be found in the region of homogeneous plastic strains. MBN along the direction of tensile stress gently decreases for higher strains, compensated by a moderate growth in the perpendicular direction (more pronounced in the case of samples cut along TD). Three counteracting effects should be considered. MBN tends to grow along the tensile stress, which is compensated by the increasing opposition of the matrix containing a higher dislocation density as a result of strain hardening. Moreover, DWs tend to be realigned towards the direction that is perpendicular to the direction of exerted tensile stress [18,19]. The growth of MBN in a direction perpendicular to the direction of the tensile load is also driven by the aforementioned effects. MBN tends to decrease due to the direction of tensile stresses (which is perpendicular), as well as due to the increasing dislocation density. On the other hand, the aforementioned DW realignment compensates these effects and prevails, as MBN in RD-TD, and especially in TD-RD, gently grows, as seen in Figure 8b.

Beyond the sample necking, the accelerated decrease in MBN can be recorded along the direction of tensile stress. Similar behaviour can also be found in perpendicular directions, but the abrupt decrease in MBN is delayed due to the contribution of the DWs’ realignment effect. The abrupt decrease in MBN in all measured directions is due to the predominating contribution of increasing dislocation density, as the MBN drops down in all directions. The role of rolling direction with respect to MBN also indicates MBN values in the region of homogenous plastic strains. MBN in RD-RD is higher compared to TD-TD, and RD-TD is lower in contrast to TD-RD.

**Figure 9:**
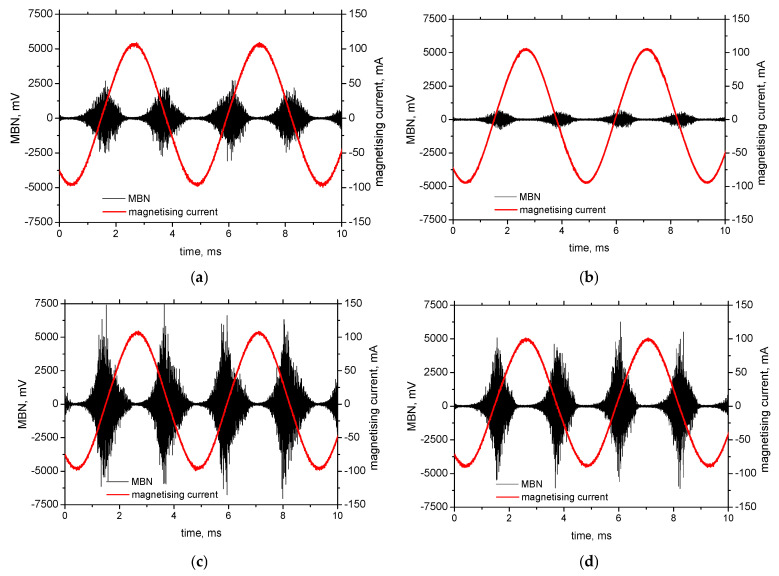
MBN signals measured ex situ as a function of *ε*; (**a**) RD-RD and *ε* = 0.5%, (**b**) RD-RD and *ε* = 21.5%, (**c**) TD-RD and *ε* = 0.5%, and (**d**) TD-RD and *ε* = 21.5%.

**Figure 10:**
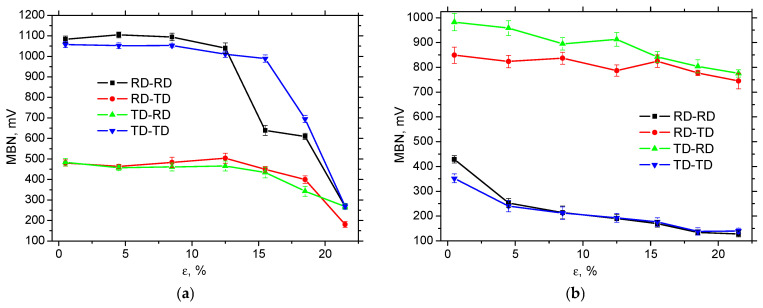
Evolution of MBN along *ε*—MicroScan (MBN in the frequency range from 10 to 1000 kHz); (**a**) in situ, and (**b**) ex situ.

The decrease in MBN at the higher strains (as depicted in Figure 8) can also be found for the MBN measured after sample unloading, see Figure 9 and Figure 10. These Figures show MBN measured over a wider frequency range, compared with Figure 8. Therefore, the higher MBN in all directions can be measured. MBN is nearly unaffected in the region of homogeneous strains, followed by a rapid descent beyond the necking. In contrast to Figure 8, the MBN along the direction of tensile stress is very similar (the same is true for the perpendicular directions). This finding suggests a specific role for low- and high-frequency MBN pulses (below 70 kHz and above 200 kHz) in creating the differences associated with MBN, as mentioned earlier and illustrated in Figure 8.

A comparison of Figure 10a,b clearly demonstrates the significant role of external tensile stresses with respect to MBN. Release of the external tensile stresses fully and remarkably reverses the MBN when comparing the MBN measured along the tensile stress and in the perpendicular direction. MBN in the direction of tensile stress abruptly drops down at the expense of very high MBN in the perpendicular directions. This behaviour is directly connected with DWs’ realignment into the direction perpendicular to the direction of tensile stress. Moreover, MBN in all directions gradually decreases due to increasing dislocation density, and this decrease is more pronounced in RD-RD and TD-TD.

**Figure 11:**
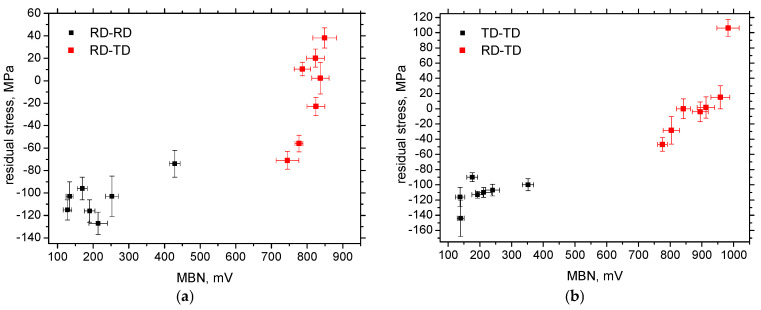
MBN versus residual stresses; (**a**) cut along RD, and (**b**) cut along TD.

The influence of external stresses on MBN is remarkable, especially in regions of elastic and homogeneous strains. On the other hand, a certain contribution of the residual stresses can also be reported, as the higher ex situ measured MBN in RD-TD and TD-RD can be linked to residual stresses shifted towards the tensile region (see Figure 6 and Figure 10b). Moreover, residual stresses in all directions are shifted towards compressive ones (or a higher amplitude of compressive stresses is attained), which might contribute to the lower MBN along the more developed *ε*. Table 4, as well as Figure 11, indicates that the correlation between MBN and residual stresses is valuable in some cases. Still, contributions of further effects should be considered, such as the increasing density of pinning sites (dislocation tangles), which hinder DWs in motion (this contributes to the decreasing MBN along with *ε*). Furthermore, the realignment of DWs into RD-TD and TD-RD, respectively, makes these directions easy axes of magnetisation at the expense of the perpendicular directions and takes the main role in the remarkably higher MBN in RD-TD and TD-RD obtained for the ex situ regime.

**Figure 12:**
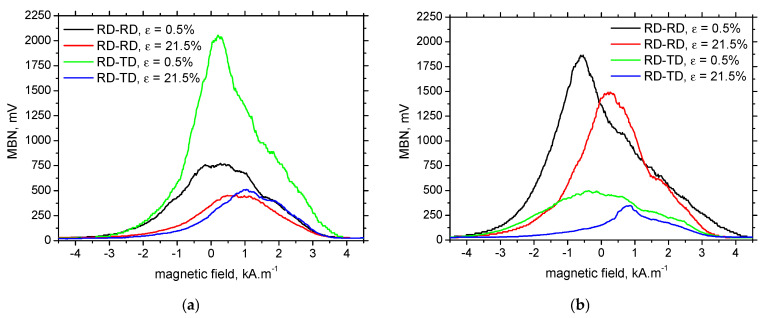
MBN envelopes as a function of loading direction and *ε*; (**a**) in situ, and (**b**) ex situ.

MBN envelopes (as those depicted in Figure 12) can be used for the extraction of *PP* as the MBN parameter linked with the magnetic, and usually also the mechanical, hardness of a body [18].

**Figure 13:**
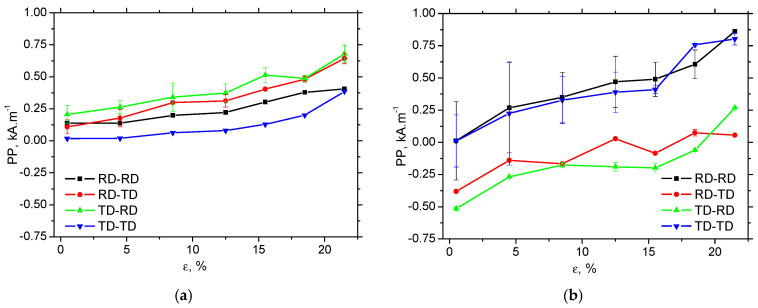
Evolution of *PP* along *ε*; (**a**) in situ, and (**b**) ex situ.

**Figure 14:**
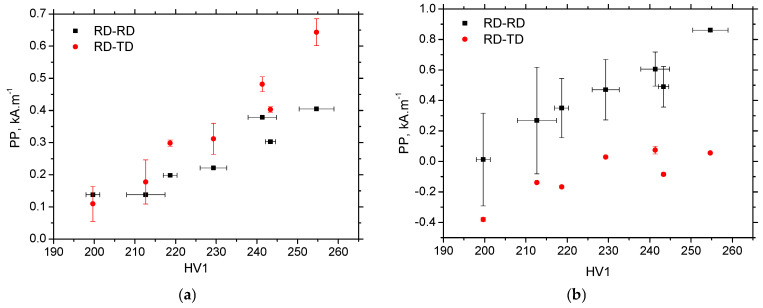
*HV1* versus *PP*; (**a**) in situ, and (**b**) ex situ.

The evolution of *PP* is mainly driven by the increasing opposition of the matrix against magnetisation, expressed in terms of an increasing density of dislocation cells, which is multiplied as a result of strain hardening, as shown in Figure 13. *PP* gradually grows with ε in both in situ and ex situ measurements. *PP* for RD-TD and TD-RD is more suitable for in situ situations, as DWs are aligned along the direction of tension, and DWs before irreversible motion must undergo a rotation phase [21]. As soon as the external stress is released, this ratio is reversed for the same reason. DWs are aligned ex situ, perpendicularly against the released tensile stress in RD-RD and TD-TD, and the corresponding *PP* is therefore greater. Figure 13 also illustrates that the differences in *PP* in the different directions are more pronounced due to the contribution of DW realignment (the ex situ regime) compared with the contribution of external tensile stresses. However, the correlation between *PP* and dislocation density, expressed in *HV1*, is strong for all directions and regimes (both ex situ and in situ), as shown in Figure 14 and Table 5. A similar correlation can also be observed between *PP* and dislocation density, as shown in Table 5.

Finally, the role of chemical composition and the corresponding microstructure on the MBN evolution and its anisotropy, as well as DW rearrangement, should be discussed. The Neel theory [38] distinguishes between the contribution of precipitates and their stress fields to the fluctuation of the spontaneous magnetic polarisation *H* as follows:

*H* = 0.167*v_o_* + 28.66*v_i_* (5)

where *v_o_* is the volume fraction of regions of the altered internal stresses and *v_i_* is the relative volume of precipitates. Being so, the growing MBN along the tensile direction after unloading can be attained for pure Fe [15] or interstitial free steels [23]. As soon as the density of lattice imperfections (especially with respect to interstitial and/or dissolved atoms) exceeds the critical threshold, the opposite evolution in MBN is obtained as reported earlier [19,21]. The microstructure of the low-alloyed steel is usually quite simple. However, the density of lattice imperfections is beyond the critical threshold (including the studied MC500), and TD emits stronger MBN after the tensile test, as contrasted against RD due to DW realignment.

## 4. Conclusions

This study revealed that the role of crystallographic and corresponding magnetic anisotropy on the evolution of MBN in regions of both elastic and plastic strains is minor. Evolution of MBN and *PP* along *ε* is driven by the superimposing contribution of stresses, dislocation density, and DWs alignment. The contribution of external stresses during tensile stressing is greater compared to residual stresses. DWs are aligned along the direction of the exerted load during tension, but their release realigns DWs into a perpendicular direction. *PP* is mainly a function of dislocation density. The role of external stress remains valuable, but less pronounced, compared to its influence on MBN. DW realignment after tensile stress release reverses MBN as well as *PP* when comparing their values measured along the tensile stress and in the perpendicular directions.

## Figures and Tables

**Table 1:** Mechanical properties of MC500 obtained from the stress–strain curves.

Yield Strength (MPa)		Ultimate Strength (MPa)		Elongation at Break (%)	
RD	TD	RD	TD	RD	TD
572 ± 10	592 ± 14	643 ± 10	653 ± 7	25 ± 1.2	25 ±1.6

**Table 2:** Chemical composition of MC500 in wt%.

Fe	C	Mn	Si	P	S	Al	Nb + Ti
bal.	0.08	1.1	0.02	0.008	0.02	0.04	0.1

**Table 3:** Nomenclature with respect to abbreviated directions used in the paper, also see Figure 1.

Abbreviation	Explanation
RD	MC500 sheet rolling direction.
TD	Transversal direction against the sheet rolling direction.
RD-RD	Sample cut along RD, loaded, and measured in RD.
RD-TD	Sample cut along RD, loaded in RD, and measured in TD.
TD-TD	Sample cut along TD, loaded, and measured in TD.
TD-RD	Sample cut along TD, loaded in TD, and measured in RD.

**Table 4:** Correlation coefficients *ρ_p_* of MBN versus residual stresses (ex situ).

Direction	RD-RD	RD-TD	TD-TD	TD-RD
	0.69	0.81	0.84	0.49

**Table 5:** Correlation coefficients *ρ_p_* of *PP* versus *HV1* and *PP* versus dislocation density.

	In Situ				Ex Situ			
Direction	RD-RD	RD-TD	TD-TD	TD-RD	RD-RD	RD-TD	TD-TD	TD-RD
*HV1*	0.94	0.96	0.96	0.96	0.96	0.86	0.97	0.93
dislocation density	-	-	-	-	0.94	0.90	0.97	0.95

## Data Availability

The original contributions presented in this study are included in the article. Further inquiries can be directed to the corresponding author.
